# Erectile dysfunction in ankylosing spondylitis patients

**DOI:** 10.1590/S1677-5538.IBJU.2016.0378

**Published:** 2017

**Authors:** Thiago Santana, Thelma Skare, Vitor Steil Delboni, Juliana Simione, Ana Paula B. Campos, Renato Nisihara

**Affiliations:** 1 Unidade de Reumatologia, Hospital Evangélico, Padre Anchieta, Curitiba, PR, Brasil;; 2 Departamento de Medicina, Universidade Positivo, Curitiba, PR, Brasil

**Keywords:** Erectile Dysfunction, Spondylitis, Ankylosing, Disease

## Abstract

**Background:**

Rheumatic diseases such as ankylosing spondylitis (AS) may be associated with sexual dysfunction.

**Aim:**

To study erectile function of a group of Brazilian AS patients comparing them with controls.

**Materials and Methods:**

This was a cross sectional study approved by the local Committee of Ethics in Research. The questionnaire IIEF (International Index of Erectile Function) was applied to 40 AS patients and 40 healthy controls. AS patients had determination of disease activity (through BASDAI or Bath Ankylosing Spondylitis Disease activity index), ASDAS (Ankylosing Spondylitis Disease Activity Score, MASES or Maastricht Ankylosing Spondylitis Score and SPARCC or Spondyloarthritis Research Consortium of Canada), function (through BASFI or Bath Ankylosing Spondylitis Functional Index and HAQ or Health Assessment Questionnaire) and BASMI (Bath Ankylosing Spondylitis Metrological Index).

**Results:**

AS patients had a median score on IIEF of 22.0 (IQR=18-25) while controls had 29 (IQR=27-30) with p<0.0001 Only 17.5% of the AS patients had no erectile dysfunction, in opposite to 87.5% of controls (p<0.0001). IIEF scores had a negative association with BASDAI (p<0.0001), HAQ (p=0.05), body mass index (P=0.03), MASES (P=0.02) and SPARCC (P=0.02) in a univariate analysis. Multiple regression showed that BASDAI was the only variable independently associated with IIEF.

**Conclusion:**

There is a high prevalence of erectile dysfunction among AS patients that is associated with disease activity measured by BASDAI.

## INTRODUCTION

Ankylosing spondylitis (AS) is a chronic rheumatic disease that affects mainly young males and belongs to the group of Spondyloarthritis (SpA) (1). The most distinguishing feature of this disease is inflammation of axial joints (starting at sacroiliac joints) causing inflammatory low back pain. Eventually, the axial disease causes joint fusion, which is associated with substantial functional impairment and with the appearance of the classical “skier posture”. Patients may also present with asymmetric oligoarthritis (especially of the lower extremities), dactylitis (sausage digits), and enthesitis (inflammation at sites of ligamentous or tendon attachment to bone). Additional features include eye and bowel inflammation, an association with preceding or ongoing infectious disorders, and a strong association with the human leukocyte antigen (HLA)-B27 (1). The diagnosis of AS, as in others SpA, can be done by the ASAS (Assessment of SpondyloArthritis International Society) classification criteria that includes a set of clinical findings such as inflammatory back pain, arthritis, enthesitis etc., presence of HLA B27 and image findings of sacroiliitis (1) (Table-1). The estimated prevalence of SpA in a Caucasian population is approximately 0.5 to 2 percent (1). Its treatment varies according to presenting clinical features but in patients with axial predominance it is based mainly on AINH (anti-inflammatory non-steroidal drugs) and biological medication such as anti-TNF alpha (TNF-α). When it affects peripheral joints or has an extra-articular involvement, sulphasalazine, methotrexate and leflunomide may be used (1).

**Table 1:** ASAS (Assessment of SpondyloArthritis international Society) Classification Criteria for Spondyloarthritis (1).

Lumbar pain >3 months in patients with age <35 years. +			
Sacroiliitis at imaging + one or more criteria bellow	or		HLA B27 + two or more criteria bellow
Inflammatory lumbar pain		Inflammatory bowel disease	
Arthritis		Good response to NSAID	
Enthesitis		Family history of Spondyloarthritis	
Dactilytis		HLA B27	
Psoriasis		Elevated C reactive protein	

Musculoskeletal chronic pain, stiffness, depression, loss of self-esteem by a disturbed perception of the own body image and fatigue are commonly seen in this disease and affect significantly the quality of life of these patients (1). Sexuality has an important role in the personal satisfaction and is associated to quality of life, being a complex aspect of human life and is more than the sexual act. Rheumatic diseases may affect all aspects of life including sexual functioning due to multifactorial disease-related factors as well as therapy. Some factors include pain, fatigue, stiffness, functional impairment, depression, anxiety, negative body image, reduced libido, hormonal imbalance and drug treatment (2). However, studies on sexual dysfunction among AS patients are contradictory; while some of them report a low level of sexual satisfaction (3-5), others do not (6, 7). A possible reason for those dissimilar outcomes is that many studies were of small size; another might be the presence of confounding factors such as duration and activity of the disease (8).

Several instruments using image and endocrinological resources have been used to identify erectile dysfunction; however, questionnaires of self-reported performance are considered an important tool to diagnose and classify this problem in clinical assays (7). The International Index of Erectile Function (IIEF) is one of such questionnaires and is considered to have high sensibility and specificity in this context (9).

In the present study, we have analyzed a sample of Brazilian AS patients aiming to know the influence of this rheumatic disease in their erectile function using the IIEF.

## MATERIALS AND METHODS

This was a cross sectional study approved by the local Committee of Ethics in Research (CAAE number 50667215.0.0000.0103) and all participants signed consent. It was a convenience sample that embraced all SpA patients that attended regular consultations in a single Rheumatologic Unit from Evangelical University Hospital in Curitiba-Paraná, Brazil, during a period of six months and that agreed to participate in the study. This sample gave a power analysis of 92%.

The sample included 40 patients with AS that fulfilled the ASAS criteria for the disease (1), older than 18 years. ASAS criteria are shown in Table-1.

As comparison group, 40 voluntary healthy men, paired for age, tobacco exposure and comorbidities answered the questionnaire.

Data on disease duration, epidemiological and clinical profile, HLA-B27 positivity was collected in medical records of AS patients. They had also determination of overall disease activity through BASDAI (Bath Ankylosing Spondylitis Disease Activity Index) (1), ASDAS (Ankylosing Spondylitis disease Activity Score) (1), erythrocyte sedimentation rate (ESR) and C reactive protein (CRP); enthesitis activity (through MASES or Maastricht Ankylosing Spondylitis Score (10) and SPARCC or Spondyloarthritis Research Consortium of Canada) (11); function through BASFI (Bath Ankylosing Spondylitis Functional Index) (1), HAQ (Health Assessment Questionnaire) (12) and BASMI or Bath Ankylosing Spondylitis metrological index (1).

Patients and controls were submitted to the IIEF. About erectile function, this questionnaire has six closed questions that were answered by patients without interference from other person or researchers. Each question has a value ranging from 1 to 5, and responses with low values represent poor condition of the quality of erectile function and those with results lower than 6-10 points were considered to have severe erectile dysfunction; values between 11 and 16 with severe moderated, values ranging from 17 to 21 as moderate dysfunction, values of 22 to 25 as mild dysfunction and those with values higher than 26 as no erectile dysfunction (8, 13) (Table-2).

**Table 2:** The International Index of Erectile Function (IIEF-5) Questionnaire (8, 13).

Over the past 6 months					
1 - How do you rate your confidence that you could get and keep an erection?	**Very low**	**Low**	**Moderate**	**High**	**Very high**

	(1)	(2)	(3)	(4)	(5)
2 - When you had erections with sexual stimulation, how often were your erections hard enough for penetration?	**Almost never/never**	**A few times (much less than half the time)**	**Sometimes (about half the time)**	**Most times (much more than half the time)**	**Almost always/always**

	(1)	(2)	(3)	(4)	(5)
3 - During sexual intercourse, how often were you able to maintain your erection after you had penetrated (entered) your partner?	**Almost never/never**	**A few times (much less than half the time)**	**Sometimes (about half the time)**	**Most times (much more than half the time)**	**Almost always/always**

	(1)	(2)	(3)	(4)	(5)
4 - During sexual intercourse, how difficult was it to maintain your erection to completion of intercourse?	**Extremely difficult**	**Very difficult**	**Difficult**	**Slightly difficult**	**Not difficult**

	(1)	(2)	(3)	(4)	(5)
5 - When you attempted sexual intercourse, how often was it satisfactory for you?	**Almost never/never**	**A few times (much less than half the time)**	**Sometimes (about half the time)**	**Most times (much more than half the time)**	**Almost always/always**

	(1)	(2)	(3)	(4)	(5)

Data was organized in contingency and frequency Tables. Normality distribution was assessed by the Kolmogorov Smirnov test. Central tendency was expressed in mean and standard deviation (SD) in parametric samples and median and interquartile intervals (IIQ) in the non-parametric data. Association studies of nominal data were done through by Fisher Exact test and by Mann Whitney test, when data was numeric. Correlation analysis of IIEF with disease activity, functional, metrological indexes, BMI and disease duration were done by the Pearson test. All variables with P<0.1 were studied through multiple regression to verify its independency. The adopted significance was 5% (P≤0.05).

## RESULTS

### A) Comparison of AS patients with controls

In the AS group patients age ranged from 24 to 63 years old (mean 45.8±11.41); 7.6% were smokers and 7.6% had arterial hypertension. In the control group, age went from 24 to 63 years (mean 46.0±11.1) none was smoker or had hypertension. Pairing showed p=0.92 for age and 0.11 for smoking and 0.11 for arterial hypertension. None of participants (patients or controls) had diabetes mellitus. In the AS group, 90% had lumbar pain; 77.5% had peripheral arthritis; 45% had anterior uveitis, 37.5% had enthesitis; 12.5% had coxalgia; 12.5% had dactylitis; 70.5% were HLA B27 positive; 32.5% were on treatment with sulphasalazine; 7.5% were on non-steroidal anti-inflammatory drugs and 7.5% on anti-TNF medications.

### b) Study of IIEF among AS patients

IIEF score in AS patients had median value of 22.0 points (IQR=18-25, range 14-30); controls had median value of 29 points (IQR=27-30, range 13-30); with p<0.0001 (Mann Whitney test).

Table-3 displays the scores of studied patients and controls according to IIEF classification and shows a significant difference between the two groups.

**Table 3:** International Index of Erectile Function (IIEF) scores in patients with Ankylosing Spondylitis (AS) and healthy controls studied.

IIEF Classification (points)	AS PATIENTS n=40	HEALTHY CONTROLS n=40	P
No erectile dysfunction (>26)	7 (17.5%)	35 (87.5%)	<0.0001
Mild (22 to 25)	13 (32.5%)	4 (10.0%)	0.02
Moderate (11 to 21)	15 (37.5%)	1 (2.5%)	0.0001
Severe (<6 to 10)	5 (12.5%)	0	0.054

Data on inflammatory, functional, laboratory measurements as well as its correlation with IIEF values is shown in Table-4. In this table, it is possible to note a negative correlation of the IIEF score with BMI (body mass index), with several indexes of inflammatory activity such as BASDAI, MASES, SPARCC and with a functional index: the HAQ.

**Table 4:** Data on clinical profile, inflammatory, functional and laboratory of Ankylosing Spondylitis patients and its correlation with IIEF (International Index of Erectile Function).

Variable	Sample Range	Correlation with IIEF		

		Pearson’s Rho	95% CI	p
Age (years)	14 - 30 (mean=21.6±4.3)	-0.16	-0.46 to 0.16	0.31
BMI (kg/m^2^)	17 - 43.7 (mean=7.8±5.2)	-0.34	-0.59 to -0.02	0.03
Disease duration (years)	2 - 26; (median 18.0; IQR=8.2-20.0)	0.25	-0.06 to 0.53	0.11
ESR (mm)	2 - 58 (mean=26.5±21.4)	0.12	-0.19 to 0.42	0.44
CRP (mg/dL)	0 - 58 (median=6.7;IQR=3.5-17.2)	0.07	-0.25 to 0.37	0.66
BASDAI	0-7.8 (median=2.4; IQR=1.4-4.0)	-0.61	-0.77 to -0.37	<0.0001
ASDAS CRP	0 - 4.7 (mean=2.1±1.0)	-0.30	-0.56 to 0.01	0.057
MASES	0 - 13.0 (median=0; IQR=0-2.0)	-0.10	-0.59 to -0.03	0.02
SPARCC	0 - 12.0; (median=0;IQR=0.0-1.5)	-0.34	-0.59 to -0.04	0.02
BASFI	0 - 9.2; (mean=3.5±2.7)	-0.21	-0.49 to 0.10	0.18
HAQ	0 - 2.4 (median=0.6;IQR=0.1-1.0)	-0.31	-0.57 to -0.0003	0.05
BASMI	0.7 - 7.2; (mean 4.0±1.9)	-0.10	-0.40 to 0.21	0.52

**BMI =** Body mass index; **ESR =** Erythrocyte sedimentation rate; **CRP =** C reactive protein; **BASDAI =** Bath Ankylosing Spondylitis index; **ASDAS =** Ankylosing Spondylitis disease Activity Score; **MASES =** Maastricht Ankylosing Spondylitis Score; **SPARCC =** Spondyloarthritis Research Consortium of Canada; **BASFI =** Bath Ankylosing Spondylitis Functional Index; **HAQ =** Health Assessment Questionnaire; **BASMI =** Bath Ankylosing Spondylitis metrological index.

All data with p<0.1 in the univariate analysis were studied through multiple regression to evaluate their independency; only BASDAI kept its correlation with IIEF (p=0.007; t=-2.86).

## DISCUSSION

Our results endorse the finding that AS male patients had worse erectile function than the general population. Most AS patients are young adults, in their active stage of sexual activity, so this finding may contribute significantly to loss of life quality in this population (14). However, despite of the fact that that only a small amount of patients (less than 20%) had normal sexual performance, most of health care providers neglect or are not prepared to discuss such problems with their patients in daily practice.

The present work also shows that inflammatory disease activity, measured by BASDAI but not by ESR and CRP is associated with sexual impairment. ESR and CRP are usually used to judge inflammatory activity in rheumatic diseases (1); nevertheless, it is well known that no serological marker is good enough to reflect the ongoing inflammation in the SpA such as AS (1) and this may justify our findings. BASDAI is a composite index that takes into account pain in spina, peripheral joints and enthesis, fatigue as well as degree and duration of morning stiffness. All of these domains are measured according to the patient’s point of view (1). Fatigue and pain are well known to hamper sexual function in patients with rheumatic diseases (5). Pirildar et al. (15) found that the duration of morning stiffness was the only clinical feature related to erectile dysfunction in AS patients. Some authors have suggested that high levels of pro-inflammatory cytokines, specifically TNF-α, involved in the pathogenesis this disease, are related to fatigue. In this context, anti-TNF drugs could help improve fatigue (14, 16) and consequently sexual function. Corroborating this idea, Matos et al. (17), studying 383 males without rheumatic disease, found a significant association between high levels of serum TNF-α and erectile dysfunction. Hotston et al. (18) have observed that these drugs could inhibit the TNF-α upregulation of phosphodiesterase type 5 expression which, in turn, impairs nitricoxide-induced pro-erectile effects, resulting in dysfunction. Our sample of anti-TNF users (n=3) was too small to allow any conclusion of the effect of this drug on sexual performance, but all users had relatively high scores (20, 25 and 27 points). Dong et al. (14), comparing 22 AS patients on anti-TNF drugs with 20 without it, found that patients on this treatment had improvement in their score of sexual quality of life that was correlated with the degree of improvement in the BASDAI.

Interesting, age, a recognized variable associated with lower sexual function (19, 20) did not show any association in our study. When men aged 18-39 years were compared with those aged 60-69, they presented 2.2 higher risk of erectile dysfunction. The same was observed by other authors (5). It is possible that the low sexual performance of young AS patients due to disease activity blunted this connection. Ireland et al. (21) described erectile dysfunction as a rare side effect of sulphasalasine (<0.1%). In our study, among 13/40 (32.5%) in use of this drug, there was no significant difference in the scores of these patients compared with others (data not shown).

In addition, it is worthwhile to observe that Dincer et al. (3) found a relationship of sexual dysfunction with lower scores in questionnaires of function, as we did with the HAQ as observed in Table-4. However, in our study, this association did not sustain itself in the multi-variated analysis, suggesting that the inflammatory components such as pain, fatigue and stiffness impair function and underlie this association. Nowadays, with the modern treatment strategies that propose aggressive treatment of inflammation, patients have a higher chance to preserve their mobility. So, loss of function due to loss of mobility seems not to exercise important influence.

In summary, we have found that AS patients have high levels of erectile dysfunction and that disease activity measured by BASDAI is associated with sexual impairment. Health care providers should be aware that sexual impairment is a consequence of AS disease activity and discuss this issue with their patients and provide referral to specialists when appropriate.
